# Revisiting the Metabolism and Bioactivation of Ketoconazole in Human and Mouse Using Liquid Chromatography–Mass Spectrometry-Based Metabolomics

**DOI:** 10.3390/ijms18030621

**Published:** 2017-03-13

**Authors:** Ju-Hyun Kim, Won-Gu Choi, Sangkyu Lee, Hye Suk Lee

**Affiliations:** 1 Drug Metabolism and Bioanalysis Laboratory, College of Pharmacy, The Catholic University of Korea, 43 Jibong-ro, Wonmi-gu, Bucheon-si, Gyeonggi-do 14662, Korea; jhyunkim@catholic.ac.kr (J.-H.K.); cwg0222@catholic.ac.kr (W.-G.C.); 2 BK21 Plus KNU Multi-Omics based Creative Drug Research Team, College of Pharmacy, Research Institute of Pharmaceutical Sciences, Kyungpook National University, Daegu 41566, Korea; sangkyu@knu.ac.kr

**Keywords:** ketoconazole, metabolite profiling, bioactivation, metabolomics

## Abstract

Although ketoconazole (KCZ) has been used worldwide for 30 years, its metabolic characteristics are poorly described. Moreover, the hepatotoxicity of KCZ limits its therapeutic use. In this study, we used liquid chromatography–mass spectrometry-based metabolomics to evaluate the metabolic profile of KCZ in mouse and human and identify the mechanisms underlying its hepatotoxicity. A total of 28 metabolites of KCZ, 11 of which were novel, were identified in this study. Newly identified metabolites were classified into three categories according to the metabolic positions of a piperazine ring, imidazole ring, and *N*-acetyl moiety. The metabolic characteristics of KCZ in human were comparable to those in mouse. Moreover, three cyanide adducts of KCZ were identified in mouse and human liver microsomal incubates as “flags” to trigger additional toxicity study. The oxidation of piperazine into iminium ion is suggested as a biotransformation responsible for bioactivation. In summary, the metabolic characteristics of KCZ, including reactive metabolites, were comprehensively understood using a metabolomics approach.

## 1. Introduction

**Figure 1:**
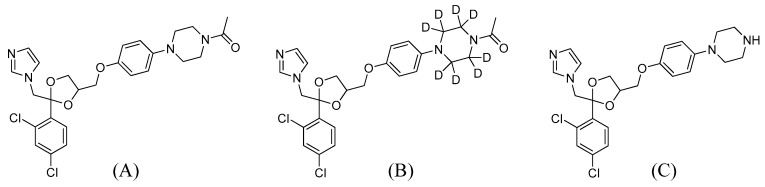
Chemical structures of (**A**) ketoconazole; (**B**) ketoconazole-d8; and (**C**) *N*-deacetyl-ketoconazole.

Ketoconazole (KCZ; Figure 1A), an imidazole-containing antifungal drug, was approved by the US Food and Drug Administration (FDA) in 1981 as the first orally available azole antifungal agent [1]. Like other azole antifungal agents, KCZ exerts its pharmacological effects by blocking the biosynthesis of ergosterol, a vital component of fungal cytoplasmic membranes, through the inhibition of lanosterol 14α-demethylase [2,3]. Owing to its broad-spectrum antifungal activity towards coccidioidomycosis, oral candidiasis, and histoplasmosis [3], KCZ is widely used in the clinic and in research.

Surprisingly, despite the use of KCZ worldwide for 30 years, its metabolic characteristics are poorly described. At the time of its approval, KCZ was suggested to be extensively metabolized into a large number of metabolites in human and mouse [4,5]. However, little evidence supports previously proposed biotransformations of KCZ, such as oxidation; scission of the imidazole, dioxolane, and piperazine rings; *O*-dealkylation; and aromatic hydroxylation. In the 1980s, trace quantities of glucuronic acid and sulfate conjugates were reported in rat excreta [6]. Remarkable progress in the understanding of KCZ metabolism was made beginning in the 1990s [7,8,9,10,11,12]. Whitehouse et al. [7,8] identified nine metabolites of KCZ in mouse liver and reported that *N*-deacetylation was the primary metabolic pathway. Rodriguez et al. [9,10] demonstrated further metabolism of *N*-deacetyl-KCZ into dialdehyde, and identified the responsible enzyme as a flavin-containing monooxygenase (FMO). The majority of suggested biotransformations were examined in 2009 by liquid chromatography-high resolution mass spectrometry (LC-HRMS) [11]. Using human and rat liver microsomes, Fitch et al. [11] proposed 16 metabolites, 10 of which were novel. Many of the previously suggested metabolic reactions—including imidazole cleavage, piperazine oxidations, with or without amide cleavage, aromatic oxidation, oxidation, and reduction—were observed; however, other biotransformations, such as *O*-dealkylation, were not identified. Also, uridine 5′-diphospho-glucuronosyltransferase (UGT) 1A4 was identified as the enzyme responsible for the glucuronidation of KCZ [13].

Drug-induced liver injury (DILI) is one of the most underlying mechanisms giving rise to drug withdrawal [14]. DILI can be categorized into three types, such as hepatocellular, cholestatic, and mixed type based on serum biochemistry [15] and several causality assessment methods have been proposed including the Roussel Uclaf Causality Assessment Method (RUCAM) [16,17]. Hepatotoxicity induced by KCZ remains the primary drawback of its therapeutic use [1]. KCZ-induced hepatotoxicity is classified as hepatocellular type [14,18] and the incidence of KCZ-induced acute liver injury ranges from 0.05% to 4.2% [19,20,21]. Although hepatic injury is usually asymptomatic and reversible, careful monitoring of patients taking KCZ is required because its toxicity is dose-independent and can be idiosyncratic. Idiosyncratic toxicity refers to rarely (generally <0.1%) and unpredictably developed toxicity and has become a major concern in drug development and clinical use. Finally, the FDA and European Medicines Agency announced new regulations for oral KCZ usage on 26 July 2013 (available on: http://www.fda.gov/Drugs/DrugSafety/ucm362415.htm and http://www.ema.europa.eu/ema/index.jsp?curl=pages/news_and_events/news/2013/07/news_detail_001855.jsp&mid=WC0b01ac058004d5c1). Unfortunately, KCZ-induced hepatic injury is not mechanistically understood (available on: https://livertox.nih.gov///Ketoconazole.htm). Limited information is available on mechanisms such as covalent binding, glutathione (GSH) depletion, and bioactivation [22,23]. Bioactivation refers to metabolic activation of xenobiotics into reactive or toxic metabolites, and can result in drug-induced toxicity [14]. Therefore, effort has focused on the assessment of bioactivation of drug candidates to avoid or manage potential risk during drug development [24,25,26,27]. The most widely used method for detection of reactive metabolites is the addition of trapping agents to microsomes. For KCZ, cyanide adducts of KCZ and hydroxy-KCZ have been identified [11,28,29]. However, the formation of GSH adducts is controversial [11,26,28,29], and other types of adducts, such as semicarbazides, were not formed [29].

Metabolomics-based non-targeted approaches are now applied in drug metabolism studies [30]; this has been reviewed recently [31,32]. By monitoring as many metabolites as possible without emphasis on particular metabolic pathways, metabolomics-guided drug metabolism studies enhance our understanding of drug metabolites, including reactive metabolites.

In this study, we evaluated the metabolic characteristics and the potential for bioactivation of KCZ. Using high resolution mass spectrometry (HRMS) and metabolomics, we enhanced our understanding of KCZ metabolic pathways and identified biologically reactive metabolites as candidates for the mechanistic assessment of hepatotoxicity.

## 2. Results

### 2.1. Overall Strategy for KCZ Metabolite Profiling

**Figure 2:**
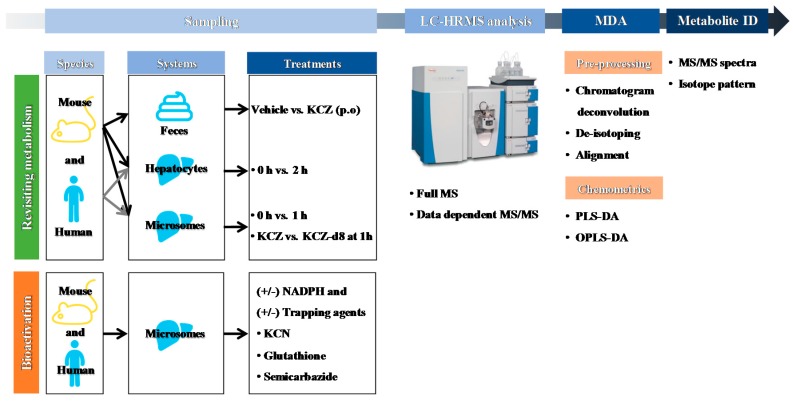
Schematic workflow for the metabolite profiling of ketoconazole using liquid chromatography–high resolution mass spectrometry (LC-HRMS)-based metabolomics. KCZ: ketoconazole, NADPH: reduced form of nicotinamide adenine dinucleotide phosphate, KCN: potassium cyanide, MS/MS: tandem mass spectrometry, MDA: multivariate data analysis, PLS-DA: partial least squares discriminant analysis, OPLS-DA: orthogonal partial least squares discriminant analysis.

A schematic workflow is presented in Figure 2. To identify an appropriate animal model for studying the metabolism and toxicity of KCZ, mouse and human were compared. After in vitro or in vivo sampling, samples were appropriately prepared and profiled using LC-HRMS. The acquired MS spectra were processed for chemometric analysis as described in Section 4.7, and meaningful variables were extracted by partial least squares discriminant analysis (PLS-DA) or orthogonal partial least squares discriminant analysis (OPLS-DA). A loading S-plot generated by OPLS-DA revealed the variables that contributed most to the separation of each group; these were considered possible KCZ metabolites and their related ions. Moreover, KCZ and KCZ-d8 were incubated separately in microsomes to enhance structural elucidation of KCZ metabolites. The ions that contributed most to separation were distributed symmetrically in the loading scatter plot and metabolite profiling focused on these top-ranking ions.

**Figure 3:**
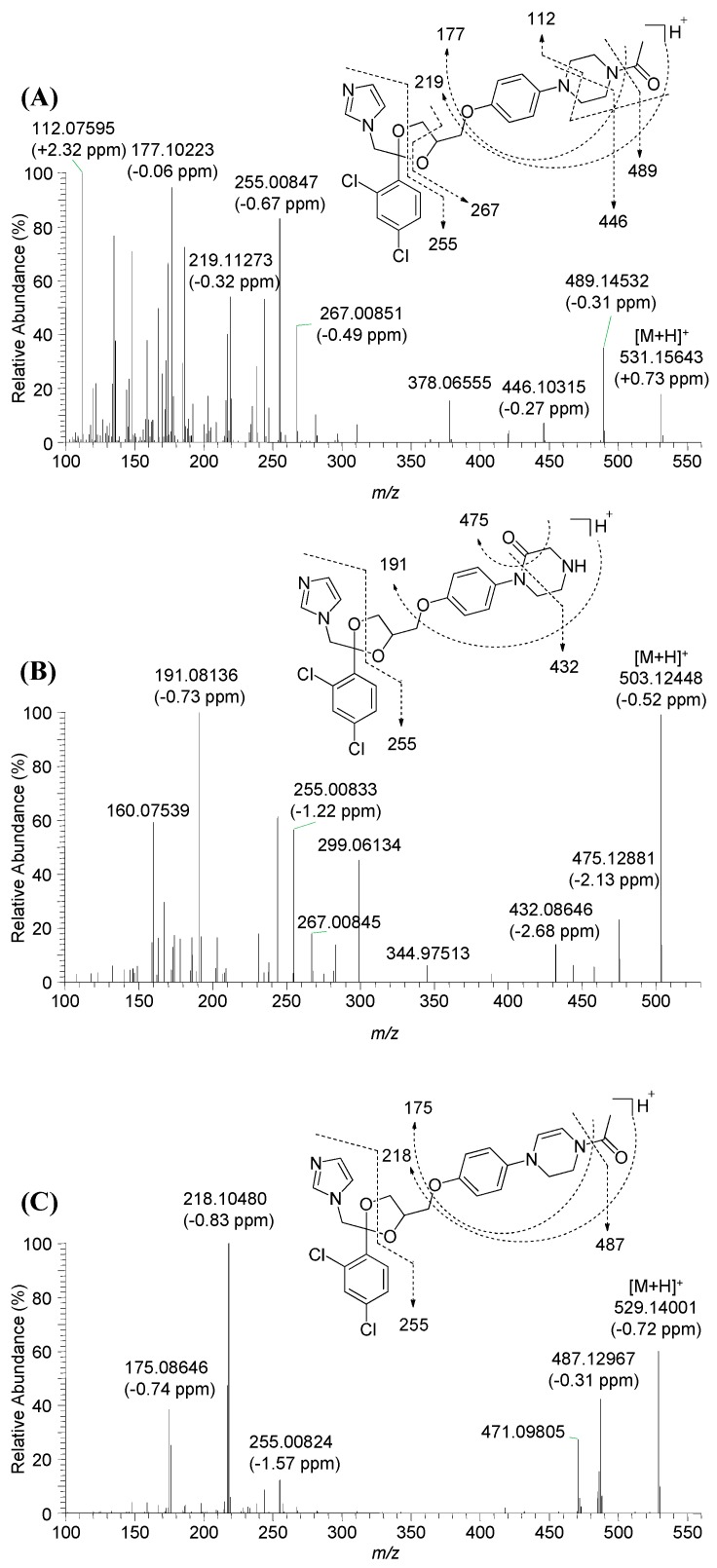

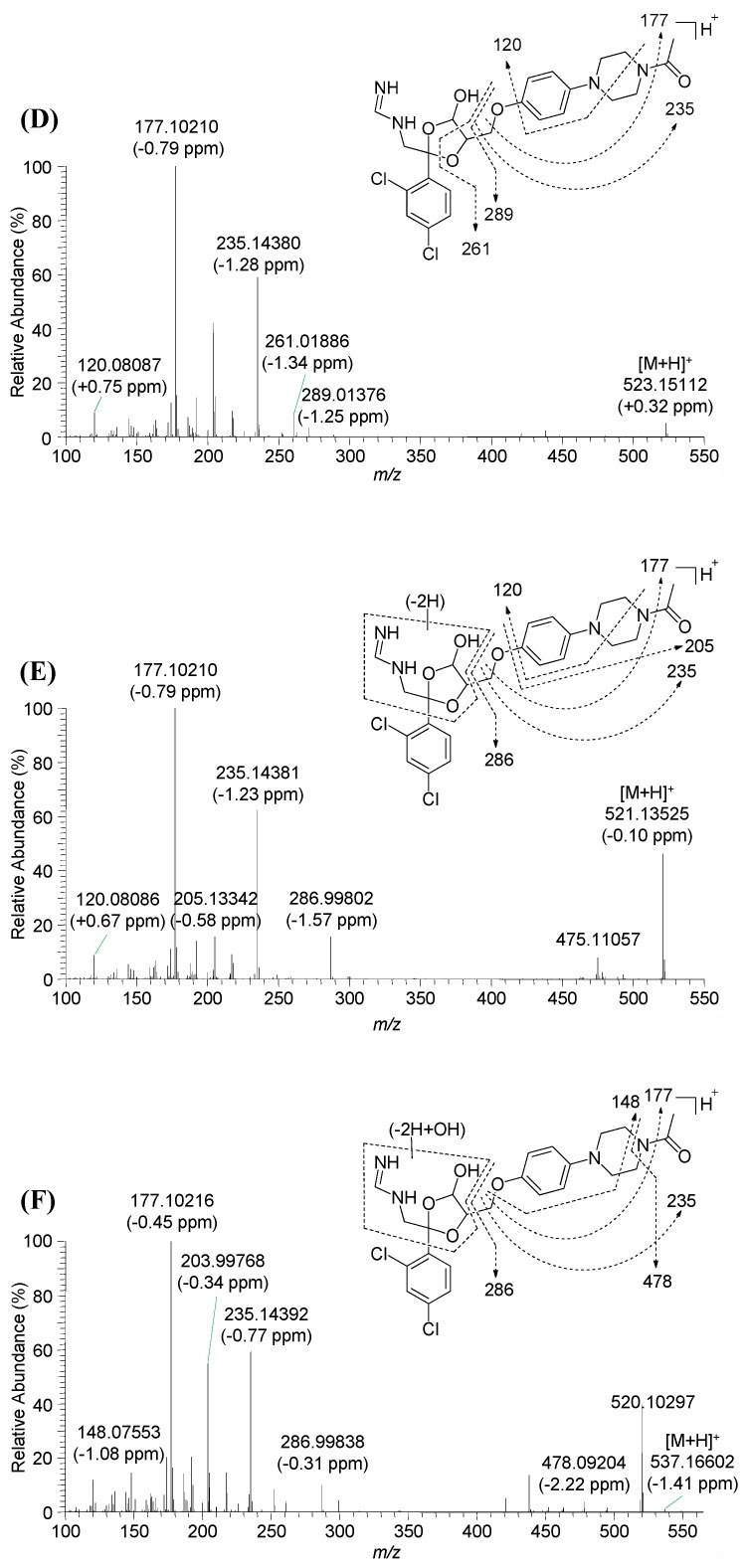
MS/MS spectra and ion chemistries of ketoconazole and newly identified metabolites. (**A**) Ketoconazole; (**B**) M17; (**C**) M20; (**D**) M22; (**E**) M27; and (**F**) M28.

In the MS spectra, KCZ exhibited three characteristic molecular ions—[M + H]^+^ at *m*/*z* 531.1563, [M + Na]^+^ at *m*/*z* 553.1378, and [M + 2H]^2+^ at *m*/*z* 266.0818—and displayed a unique isotope distribution pattern with a relative abundance ratio of 10:7:1 and a 2 amu difference, caused by two atoms of chlorine. These properties provided additional information for identification of KCZ metabolites from the complex mass spectral background. KCZ produced several characteristic fragment ions at *m*/*z* 489.1448, *m*/*z* 446.1028, *m*/*z* 267.0082, *m*/*z* 255.0082, *m*/*z* 219.1125, *m*/*z* 177.1021, and *m*/*z* 112.0757 (Figure 3A). These ions served as diagnostic product ions for the structural elucidation of KCZ metabolites. Indeed, *m*/*z* 255.0082 was proposed as 1-(2,4-dichlorophenyl)-1-hydroxy-2-(1*H*-imidazol-1-yl)ethan-1-ylium ion and played a key role in the identification and structural elucidation of metabolites by providing information on whether biotransformation involved the dichlorophenyl imidazole moiety of KCZ. Finally, the chemical structures of metabolites were investigated using their product ions and determined by using Mass Frontier software.

### 2.2. Identification of KCZ Metabolites

Metabolomics-guided metabolite profiling from mouse and human resulted in 28 metabolites including 11 newly identified ones. The chemical formulae, molecular ions, mass accuracies, retention time, and identified samples are summarized in Table 1.

#### 2.2.1. Mouse and Human Liver Microsomes

The OPLS-DA score plot clearly indicated a difference between the 0 and 1 h incubation groups, and the loading S-plot generated by OPLS-DA revealed several possible KCZ metabolites and their related ions that contributed the most to the separation (Figure S1A). The PLS-DA loading scatter plot from the microsomal incubation of KCZ and KCZ-d8 showed several possible metabolite ions (Figure S1B).

Overall, 20 metabolites of KCZ were identified in mouse liver microsomes, including six novel metabolites (Figure S2). M1 was observed at *m*/*z* 565.1625 ([M+H]^+^ ion, 34 amu higher than KCZ) and produced fragment ions at *m*/*z* 463.1186, *m*/*z* 421.1080, *m*/*z* 277.1547, *m*/*z* 259.1441, *m*/*z* 203.9978, *m*/*z* 177.1022, and *m*/*z* 112.0757 (Figure S3A). Based on the presence of ions at *m*/*z* 421.1080, *m*/*z* 117.1022, and *m*/*z* 112.0757, and the absence of *m*/*z* 255.0086, M1 was suggested to be a metabolite from biotransformation at the dichlorophenyl imidazole moiety. M1 has been reported to be an oxidative metabolite at the imidazole moiety [11].

M2 was observed at *m*/*z* 507.1565 (24 amu less than KCZ), indicating loss of C_2_ from KCZ. M2 yielded fragment ions at *m*/*z* 479.1247, *m*/*z* 235.1441, *m*/*z* 231.0086, *m*/*z* 212.9981, and *m*/*z* 112.0757 (Figure S3B). Based on the presence of an ion at *m*/*z* 112.0757 and the absence of *m*/*z* 255.0086, M2 was also suggested as a metabolite from biotransformation at the dichlorophenyl imidazole moiety. This was supported by the presence of fragment ions at *m*/*z* 231.0086, which was 24 amu less (loss of C_2_) than *m*/*z* 255.0086. In line with a previous report [11], M2 was suggested to be a metabolite generated by loss of two carbons from the imidazole moiety.

M3 was observed at *m*/*z* 517.1045 (14 amu less than KCZ) and produced fragment ions at *m*/*z* 255.0086 and *m*/*z* 245.0921 (Figure S3C). M3 was suggested to be a deacetylated and piperazine-oxidized metabolite.

M4, M5, M6, and M7 were observed at *m*/*z* 547.1510 (16 amu higher than KCZ) with retention times of 6.2, 7.3, 7.8, and 9.2 min, respectively, and indicating hydroxy-KCZ. M4 and M7 were too low to acquire their MS/MS spectra. M4, M5, and M7 were observed at *m*/*z* 555.2012 (8 amu higher than 547), whereas M6 was observed at *m*/*z* 554.1949 (7 amu higher than 547) after incubation of KCZ-d8 in mouse liver microsomes. These results indicated that hydroxylation of M4, M5, and M7 took place outside of the piperazine ring, whereas that of M6 occurred at the piperazine ring. As a representative of hydroxy-KCZ, the fragment ions of M5 was shown in Figure S4A.

M8 was observed at *m*/*z* 505.1404 (26 amu less than KCZ), indicating loss of C_2_H_2_ from KCZ. M8 produced fragment ions at *m*/*z* 487.1298, *m*/*z* 463.1298, *m*/*z* 446.1033, *m*/*z* 255.0086, and *m*/*z* 122.0600 (Figure S4B). The presence of *m*/*z* 463.1298 (26 amu less than 489.1455) and *m*/*z* 255.0086 indicated that C_2_H_2_ was lost from *N*-deacetyl-KCZ, without a metabolic change in the dichlorophenyl imidazole moiety. Therefore, M8 was suggested as a metabolite with opened piperazine ring.

M9 was observed at *m*/*z* 257.0244, indicating the dichlorophenyl imidazole moiety via the scissoring of dioxolane ring. M9 produced fragment ions at *m*/*z* 188.9869, 153.0102, and 125.0153 which are corresponding to 2-(2,4-dichlorophenyl)-2-hydroxyethan-1-ylium, 2-chloro-5-(1-hydroxyvinyl)benzene-1-ylium, and 5-chloro-2-methylbenzene-1-ylium ion, respectively (Figure S4C).

M10 was observed at *m*/*z* 545.1364 (14 amu higher than KCZ). Fragment ions of M10 were observed at *m*/*z* 503.1247, *m*/*z* 473.1142, *m*/*z* 432.0876, *m*/*z* 267.0086, *m*/*z* 255.0086, and *m*/*z* 191.0815 (Figure S5A). Together with the alteration of *m*/*z* 255.0086, the presence of *m*/*z* 503.1247 and *m*/*z* 191.0815 (which were 14 amu higher than *m*/*z* 489.1455 and *m*/*z* 177.1022, respectively), oxidative biotransformation in the piperazine ring was suggested.

M11 was observed at *m*/*z* 489.1459 (42 amu less than KCZ) with a retention time of 3.6 min, and identified as *N*-deacetyl-KCZ using an authentic standard (Figure 1C). M11 produced fragment ions at *m*/*z* 446.1033, *m*/*z* 267.0086, *m*/*z* 255.0086, *m*/*z* 178.1101, and *m*/*z* 136.0757 (Figure S5B). These ions were used in the structural elucidation of KCZ metabolites.

M12 and M13 were observed at *m*/*z* 420.0876 with retention times of 1.8 and 2.2 min, respectively. These two metabolites were calculated as C_20_H_19_Cl_2_N_3_O_3_. Based on previous reports [8,11], M12 and M13 were suggested to be isomers of completely oxidized piperazine metabolites.

M14 was observed at *m*/*z* 343.0247. Although the fragment ions of M14 were insufficient to elucidate its chemical structure, M14 could be suggested as a dichlorophenyl imidazole moiety generated by *O*-dealkylation based on the exact mass of molecular ion and a previous report [11].

M15 was observed at *m*/*z* 517.1409 (14 amu less than KCZ). The fragment ions were observed at *m*/*z* 489.1455, *m*/*z* 421.1080, *m*/*z* 267.0086, *m*/*z* 255.0086, *m*/*z* 238.0059, *m*/*z* 205.0972, and *m*/*z* 174.0913 (Figure S5C). Most of the fragment ions were identical to those of *N*-deacetyl-KCZ (M11), indicating a metabolic alteration at the terminal acetyl group. Based on a previous report [8], M15 was suggested to be *N*-formylpiperazine-KCZ.

M16 was observed at *m*/*z* 463.1308 (26 amu less than M11). The fragment ions were observed at *m*/*z* 446.1033, *m*/*z* 420.0876, *m*/*z* 267.0086, *m*/*z* 255.0086, and *m*/*z* 122.0600 (Figure S6A). Based on the presence of *m*/*z* 446.1033, M16 was suggested to be an ethylenediamine form of *N*-acetyl-KCZ [8].

M17 and M18 were observed at *m*/*z* 503.1247 with retention times of 5.9 and 6.7 min, respectively. M17 and M18 yielded identical fragment ions at *m*/*z* 475.1288, *m*/*z* 432.0865, *m*/*z* 299.0613, *m*/*z* 267.0085, *m*/*z* 255.0083, *m*/*z* 191.0814, and *m*/*z* 160.0754 (Figure 3B). Based on a previous report [11], M17 and M18 were suggested to be structural isomers of oxidized piperazine metabolites of M11.

M19 was observed at *m*/*z* 519.1204 (2 amu less than KCZ), indicating hydroxylated M17 or M18. M19 yielded fragment ions at *m*/*z* 491.1247 (16 amu higher than 475), *m*/*z* 473.1142, and *m*/*z* 255.0086 (Figure S6B). The presence of *m*/*z* 491.1247 indicated that M19 was formed via hydroxylation of the piperazine moiety; however, the hydroxylated position could not be identified.

M20 was observed at *m*/*z* 529.1404 (2 amu less than KCZ), indicating dehydro-KCZ. The fragment ions were observed at *m*/*z* 487.1297, *m*/*z* 471.0981, *m*/*z* 267.0088, *m*/*z* 255.0082, *m*/*z* 218.1048, and *m*/*z* 175.0865 (Figure 3C). The presence of *m*/*z* 175.0865 (2 amu less than 177) and *m*/*z* 255.0082 indicated that M20 was formed via dehydrogenation of the piperazine moiety.

Similar to the results from mouse liver microsomes, human liver microsomal incubates were clearly separated in the OPLS-DA score plots of the 0 and 1 h incubations. The loading S-plot revealed several possible KCZ metabolites and their related ions that contributed most to the separation (Figure S7A). The PLS-DA from human liver microsomal incubations of KCZ and KCZ-d8 were also clearly distinguishable (Figure S7B). The majority of metabolites identified in mouse liver microsomes were also detected in human liver microsomes, with the exception of M3, M12, M13, M14, M17, and M19 (Figure S8). None of the metabolites identified in liver microsomes were unique to human.

#### 2.2.2. Mouse or Human Hepatocytes

Metabolites of KCZ including phase II reaction were investigated in mouse and human hepatocytes at 0 and 2 h. The OPLS-DA score plot clearly indicated a difference between the 0 and 2 h groups, and the loading S-plot generated by OPLS-DA revealed several possible KCZ metabolites and their related ions that contributed most to the separation (Figures S1C and S7C). Two metabolites were additionally detected in mouse hepatocytes compared to liver microsomes (Figure S9).

M21 was observed at *m*/*z* 707.1876 (176 amu higher than KCZ), indicating a KCZ glucuronide. M21 yielded fragment ions at *m*/*z* 531.1560 and *m*/*z* 489.1444, which were representative product ions of KCZ (Figure S6C).

M22 was observed at *m*/*z* 523.1510 (16 amu higher than M2). The fragment ions of M22 were observed at *m*/*z* 289.0141, *m*/*z* 261.0189, *m*/*z* 235.1438, *m*/*z* 203.9976, *m*/*z* 177.1021, and *m*/*z* 120.0809 (Figure 3D). Based on the presence of fragment ions at *m*/*z* 177.1021 and *m*/*z* 235.1438, and the absence of *m*/*z* 255.0086, M22 was proposed as hydroxy-M2 via hydroxylation at dioxolane ring.

An S-plot by OPLS-DA identified possible metabolites in human hepatocytes (Figures S7C and S10), three of which (*m*/*z* 563.1459 (M23 and M24) and *m*/*z* 507.1560 (M25)) were not detected in human liver microsomes. M23 and M24 were observed at *m*/*z* 563.1459 with retention times of 10.2 and 10.4 min, respectively. These metabolites were previously reported as M5 and M6 [11]. M25 was observed at *m*/*z* 507.1560 with a retention time of 8.0 min, and was suggested to be an isobar of M2.

#### 2.2.3. Mouse Feces

**Figure 4:**
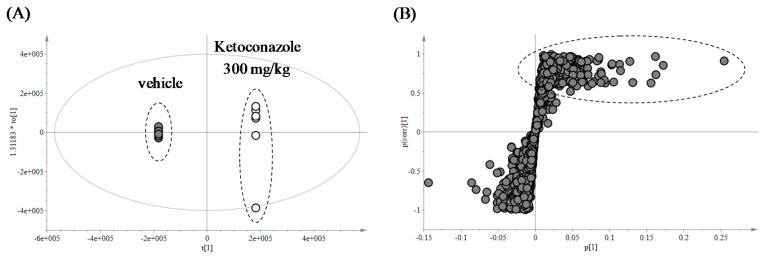
Multivariate data analysis of ketoconazole metabolites in mouse feces. (**A**) Score plot; and (**B**) loading S-plot generated by orthogonal partial least squares discriminant analysis (OPLS-DA). Top-ranking ions were highlighted with dotted ellipse.

In vivo metabolite profiling was conducted with mouse feces. As shown in Figure 4A, the OPLS-DA score plot clearly indicated differences between the vehicle- and KCZ-treated groups. The loading S-plot generated by OPLS-DA revealed several possible KCZ metabolites and their related ions that contributed most to the separation (Figure 4B). Metabolite profiling focused on these top-ranking ions.

**Figure 5:**
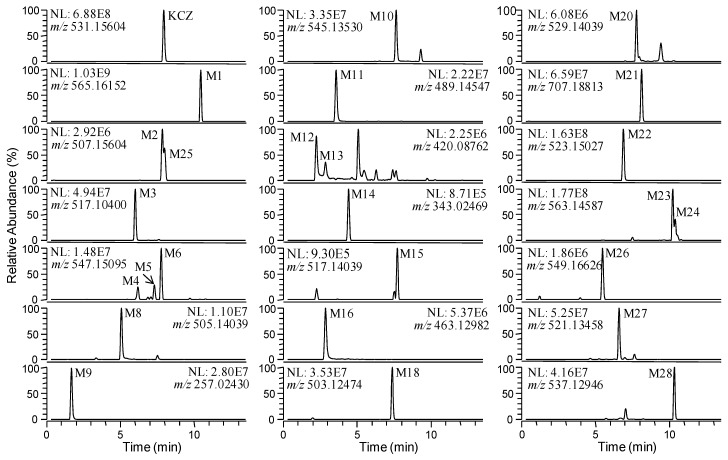
Extracted ion chromatograms of ketoconazole and its metabolites in mouse feces after oral administration of 300 mg/kg ketoconazole.

Representative extracted ion chromatograms of KCZ metabolites identified in mouse feces are shown in Figure 5. In addition to the metabolites identified in mouse liver microsomes and/or hepatocytes, three metabolites, M26–M28, were detected in mouse feces.

M26 was observed at *m*/*z* 549.1663 (18 amu higher than KCZ). M26 produced fragment ions of *m*/*z* 489.1091, *m*/*z* 446.1033, *m*/*z* 255.0086, and *m*/*z* 192.1019, indicating biotransformation at the terminal acetyl moiety (Figure S6D).

M27 was observed at *m*/*z* 521.1346 (2 amu less than M22). Product ions of M27 at *m*/*z* 120.0808, *m*/*z* 177.1022, *m*/*z* 205.1335, *m*/*z* 235.1441, and *m*/*z* 286.9985, together with the absence of *m*/*z* 255.0086, indicated biotransformation at the imidazole and dioxolane moieties (Figure 3E). Therefore, M27 was suggested to be dehydro-M22, however, the accurate dehydrogenated position could not be identified.

M28 was observed at *m*/*z* 537.1295 (16 amu higher than M27), indicating it to be hydroxy-M27. M27 produced fragment ions at *m*/*z* 478.0931, *m*/*z* 421.1080, *m*/*z* 286.9985, *m*/*z* 235.1441, *m*/*z* 203.9978, *m*/*z* 177.1022, and *m*/*z* 148.0757. By comparison with the fragment ions of M27, hydroxylation at *N*-[(4-hydroxy-1,3-dioxolan-2-yl)methyl]formimidamide was suggested (Figure 3F).

### 2.3. Identification of Reactive Metabolites

**Figure 6:**
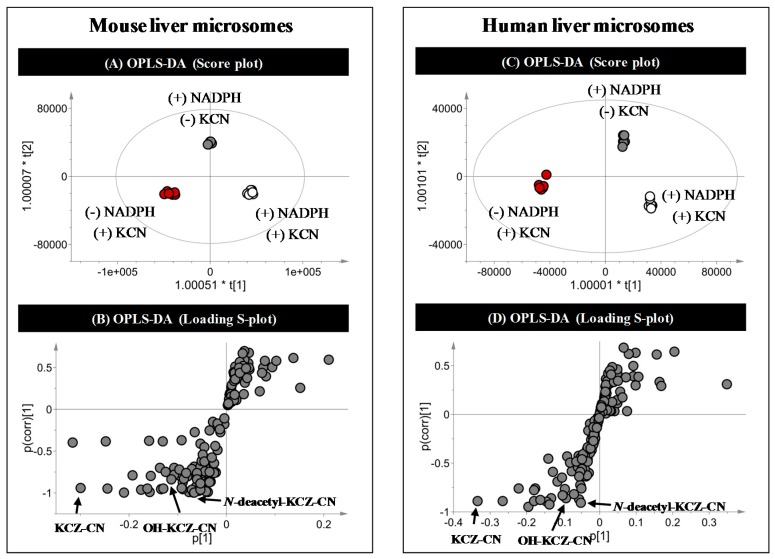
Multivariate data analysis for profiling of ketoconazole cyanide adducts. (**A**) Score plot and (**B**) loading S-plot generated by OPLS-DA from mouse liver microsomal incubations; (**C**) Score plot and (**D**) loading S-plot generated by OPLS-DA from human liver microsomal incubations.

Potassium cyanide (KCN) was used to trap reactive intermediates of KCZ in three treatment groups. Supervised OPLS-DA analysis clearly separated the three groups (Figure 6A,C). The S-plot generated by OPLS-DA analysis showed the contributions of the various ions to the separation (Figure 6B,D). No species-specific cyanide adducts of KCZ were detected.

**Figure 7:**
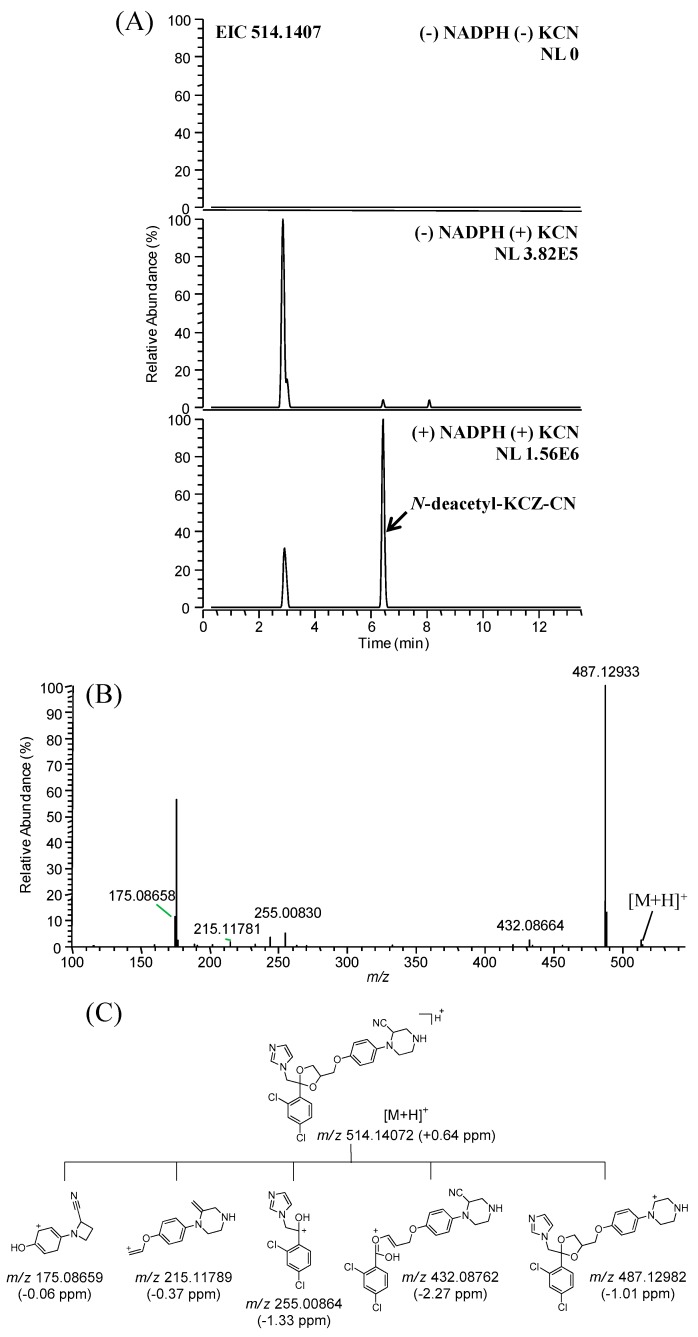
(**A**) Extracted ion chromatograms; (**B**) MS/MS spectrum; and (**C**) proposed fragment ions of cyanide adducts of *N*-deacetyl-ketoconazole.

The ion that made the greatest contribution to the separation, *m*/*z* 556.1513, was identified as a KCN adduct of KCZ [11,28,29]. Additionally, KCN adducts of hydroxy-KCZ and *N*-deacetyl-KCZ were identified at *m*/*z* 572.1462 and *m*/*z* 514.1407, respectively. Cyanide adducts of hydroxy-KCZ have been reported previously [11,28,29], whereas this is the first report of an *N*-deacetyl-KCZ adduct. The *N*-deacetyl-KCZ cyanide adduct was detected only in the presence of both NADPH and KCN (Figure 7A), and produced fragment ions of *m*/*z* 487.1298, *m*/*z* 432.0876, *m*/*z* 255.0086, *m*/*z* 215.1179, and 175.0866 (Figure 7B). Based on the product ions and the transformation of cyanide adducts to iminium ions, chemical structures for the *N*-deacetyl-KCZ cyanide adducts were proposed (Figure 7C). Neither GSH nor semicarbazide adducts of KCZ were detected.

## 3. Discussion

Establishing a comprehensive understanding of the metabolic characteristics of drugs is essential in drug development and clinical use. By studying drug metabolism in detail, dosing regimen can be determined and drug-drug interaction potentials can be mechanistically understood. The importance of metabolism is more critical when the drug can be biotransformed into biologically active or reactive metabolites. Especially, the formation of reactive metabolites is believed to be the most important underlying mechanism of idiosyncratic adverse drug reactions and is intensively studied to avoid or manage the reactive metabolite formations [24,25,26,27,33,34,35].

**Figure 8:**
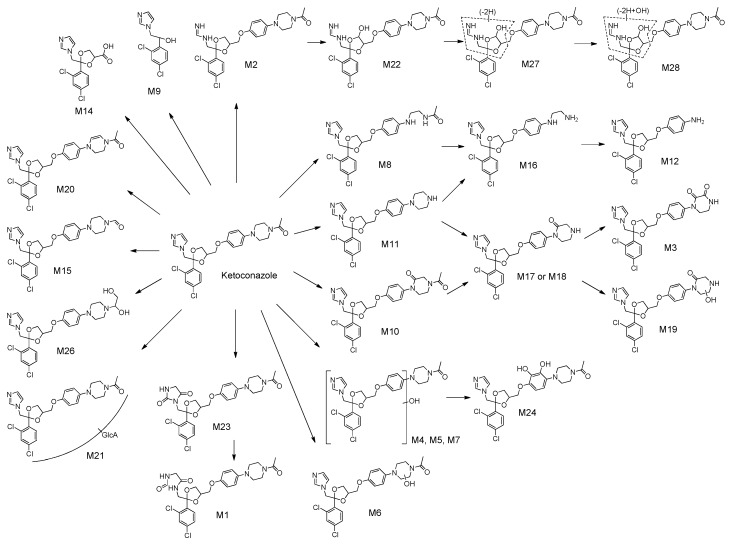
Proposed metabolic pathways of ketoconazole.

In this study, we applied metabolomics approaches to assess the metabolic pathways of KCZ, including its reactive metabolites. Eleven novel metabolites of KCZ were identified in mouse and human, together with 17 known metabolites (Figure 8). With the exception of isobars previously reported by Fitch et al. [11] (M6, M13, and M25), newly identified metabolites were classified into three categories: those with alterations at the piperazine ring (M17–M20), imidazole ring (M22, M27, and M28), or *N*-acetyl moiety (M26). M17 and M18 were suggested as isomeric metabolites of *N*-deacetyl-KCZ (M11) via oxidation of piperazine to ketone, and M19 was proposed as a hydroxylated metabolite of M17 or M18. M20 was suggested to be a metabolite of KCZ with a reduced piperazine moiety. Moreover, a series of metabolites from M2, from which two carbons were lost from the imidazole moiety, were identified: hydroxy-M2 (M22), dehydro-M22 (M27), and hydroxy-M27 (M28). M26 was identified as a metabolite of KCZ generated by reduction and hydroxylation at the *N*-acetyl moiety.

Conventionally, metabolite profiling was conducted based on previously known biotransformations. Therefore, the results were strongly empirical and dependent on the investigator’s level of knowledge. However, metabolomics-guided drug metabolism studies have not focused on specific metabolic pathways; therefore, this non-targeted approach facilitates the identification of novel metabolites, the characterization of metabolic enzymes, and the profiling of reactive metabolites [32,36,37,38,39,40,41,42]. This approach is complementary to conventional drug metabolism studies.

To investigate the metabolic characteristics of KCZ under hepatotoxic conditions, 300 mg/kg KCZ was orally administered to mice. Serum alanine transaminase and aspartate transaminase were significantly increased by 5- and 4-fold, respectively, compared to the vehicle treated group. The feces were selected as biological samples for metabolite profiling in order to fully understand metabolic pathways of KCZ regardless of sampling time. Overall, the metabolic characteristics of KCZ in human were comparable to those in mouse. As shown in Table 1, all human metabolites of KCZ were identified in mouse in in vitro or in vivo samples. Animal models are commonly used in drug development to characterize the biological behaviors of drugs and predict those in humans. From the drug metabolism and toxicology perspectives, the most concerning issue is the presence of human metabolites that were not detected in animals or were present in excess in humans compared with animals. Our work suggested that the metabolism of KCZ is similar in human and mouse; therefore, metabolic information from mouse models is relevant to humans.

The alicyclic amine in KCZ undergoes oxidation, which generates the corresponding iminium ion; this reaction is mediated by cytochrome P450 or monoamine oxidase. In addition, evidence linking the bioactivation of alicyclic amine into reactive iminium ion has been reported for several piperazine-containing drugs and xenobiotics, such as nefazodone, mianserin, and MB243 [43,44,45].

In this study, we discovered three cyanide adducts of KCZ (KCZ, hydroxy-KCZ, and *N*-deacetyl-KCZ) in human and mouse liver microsomal incubation systems (Table 2).

Among them, KCZ and hydroxy-KCZ adduct have been reported previously [11,28,29]; this is the first report of *N*-deacetyl-KCZ adduct. In particular, *N*-deacetyl-KCZ (M11), a major metabolite of KCZ, is generated by CYP3A4 and is further metabolized to the reactive dialdehyde form by FMO [7,9,10]. *N*-deacetyl-KCZ and its metabolite have been demonstrated to be more cytotoxic than KCZ [22,23]. Therefore, the formation of cyanide adducts with *N*-deacetyl-KCZ is a good “flag” for the bioactivation and related hepatotoxicity of KCZ. In contrast, GSH and semicarbazide conjugates of KCZ were not detected in vitro or in vivo. Our work is consistent with previous in vitro findings using human liver microsomes [11,26,29]. This suggested that KCZ-induced hepatotoxicity is not mainly caused by GSH depletion.

## 4. Materials and Methods

### 4.1. Chemicals and Reagents

KCZ (≥98%), Krebs–Henseleit buffer, β-nicotinamide adenine dinucleotide phosphate (NADP), the reduced form of NADP (NADPH), glucose-6-phosphate, glucose-6-phophate dehydrogenase, magnesium chloride, potassium phosphate, GSH, potassium cyanide (KCN), semicarbazide, dimethyl sulfoxide (DMSO), and methyl cellulose were purchased from Sigma-Aldrich (St. Louis, MO, USA). LiverPool™ 50-donor pooled cryopreserved human hepatocytes, InVitroGRO™ HT Medium, and InVitroGRO™ KHB were obtained from Bioreclamation IVT (Brussels, Belgium). Ketoconazole-d8 (KCZ-d8) and *N*-deacetyl-ketoconazole (*N*-deacetyl-KCZ; Figure 1B,C) were purchased from Toronto Research Chemicals (Toronto, ON, Canada). Corning^®^Gentest™ mouse cryopreserved hepatocytes, Corning^®^Gentest™ high-viability cryohepatocyte recovery medium, 50-mL conical tubes, and 96-well plates were obtained from Corning Life Sciences (Tewksbury, MA, USA). Acetonitrile, methanol, and water (LC-MS grade) were purchased from Fisher Scientific (Fair Lawn, NJ, USA). Other chemicals used were of the highest quality available.

### 4.2. In Vitro Incubation of KCZ in Liver Microsomes

KCZ (20 µM) was incubated with 1 mg/mL human or mouse microsomal protein in 50 mM potassium phosphate buffer (pH 7.4) at 37 °C for 60 min. The reaction was started by adding 2 mM NADPH in sextuplicate. KCZ-d8 (20 µM) was incubated separately to facilitate structural elucidation of KCZ metabolites. The reaction was stopped by adding 200 μL of ice-cold acetonitrile. Time 0 samples were prepared by quenching immediately after adding NADPH as a control. The reaction mixtures were then centrifuged at 15,000× *g* for 10 min at 4 °C and 180 μL of the supernatants were dried in a vacuum concentrator. The residue was reconstituted in 100 μL of 10% methanol; 5 μL aliquots were injected into the LC-HRMS system to perform metabolite profiling.

### 4.3. In Vitro Incubation of KCZ in Hepatocytes

Pooled cryopreserved human hepatocytes from 50 donors (LiverPool™) were carefully thawed according to the manufacturer’s instructions. Cryopreserved mouse hepatocytes (Corning^®^Gentest™) were purified and recovered using a High Viability CryoHepatocyte Recovery Kit in accordance with the manufacturer’s protocols. Thawed human or mouse hepatocytes were resuspended in Krebs-Henseleit buffer to a final density of 1.0 × 10^6^ cells/mL. Subsequently, 60 μL of human and mouse hepatocyte suspension and 60 μL of 40 μM KCZ solution in Krebs-Henseleit buffer were added to a 96-well plate and incubated in quadruplicate for 0 and 2 h in a humidified CO_2_ static incubator. Incubations were stopped at predetermined time points by adding 120 μL of ice-cold methanol:water (3:1, *v*/*v*) to each well, followed by 5-min sonication and centrifugation at 15,000× *g* for 10 min at 4 °C. After transferring 80 µL of supernatant into an autosampler vial, 5 µL of samples were injected into the LC-HRMS system.

### 4.4. In Vivo Metabolite Profiling of KCZ in Mouse Feces

Specific pathogen-free male ICR mice (7–8-weeks-old) were obtained from Samtaco Inc. (Osan, Korea) and acclimated for at least 1 week. The animal quarters were maintained at a temperature of 22 ± 2°C on a 12 h/12 h light/dark cycle and a relative humidity of 55% ± 5%. The study protocol was approved by the Institutional Animal Care and Use Committee (IACUC) of The Catholic University of Korea (approval No. 2015-020, approval date 30 October 2015). KCZ was suspended in a 0.5% methyl cellulose solution at 30 mg/mL, and orally administered to mice for 5-consecutive days at 10 mL/kg. Feces were collected 24 h after last dosing. Water (10 μL) was added to each 1 mg of feces, followed by vortex mixing. Following 10 min sonication, 200 μL of fecal homogenates was extracted by adding an equal volume of methanol:acetonitrile (1:1, *v*/*v*). After centrifugation at 15,000× *g* for 10 min at 4 °C, 300 μL of the supernatant was dried in a vacuum concentrator. The residue was reconstituted in 100 μL of 25% methanol and 5 μL aliquots were injected into the LC-HRMS system.

### 4.5. Screening of KCZ Reactive Metabolites in Liver Microsomes

The method used by Li et al. was adapted in this study [46]. KCZ (30 µM) was separately incubated in potassium phosphate buffer (50 mM, pH 7.4) containing human or mouse liver microsomes (1 mg/mL), NADPH (2 mM), and the trapping reagents GSH (2.5 mM), KCN (1.5 mM), or semicarbazide (2.5 mM). Reactions lacking NADPH or trapping agent served as controls. The reactions were initiated by the addition of NADPH and were stopped by the addition of 200 µL of ice-cold acetonitrile at 0 and 60 min. The reaction mixture was centrifuged at 15,000× *g* for 10 min at 4 °C and 180 μL of the supernatants were dried in a vacuum concentrator. The residue was reconstituted in 100 μL of 10% methanol and 5 μL aliquots were injected into the LC-HRMS system to screen reactive metabolites.

### 4.6. High-Performance Liquid Chromatography (HPLC)–High Resolution Mass Spectrometry (HRMS) Analysis

An Accela HPLC system coupled with a Q-Exactive Orbitrap mass spectrometer (Thermo Fisher Scientific Inc., Waltham, MA, USA) was used. A Halo^®^ C18 column (2.7 μm, 2.1 mm i.d. ×100 mm; Advanced Materials Technology, Wilmington, DE, USA) was used as the stationary phase. The HPLC mobile phases consisted of 5% methanol in 0.1% formic acid (mobile phase A; MPA) and 95% methanol in 0.1% formic acid (mobile phase B; MPB). Gradient elution of mobile phases was conducted at a flow rate of 0.4 mL/min. The gradient condition for the separation of KCZ and its metabolites was as follows: 25% MPB for 1.5 min, 25% to 60% MPB for 7.5 min, 60% to 90% MPB for 0.2 min, 90% MPB for 2.8 min, 90% to 25% MPB for 0.1 min, and 25% MPB for 2.9 min. The mass spectra were obtained using a heated electrospray ionization (HESI) source in positive mode. HESI source conditions for metabolite profiling were optimized as follows: sheath gas flow rate, 35 (arbitrary units); auxiliary gas flow rate, 10 (arbitrary units); spray voltage, 4 kV; heater temperature, 350°C. Data were acquired using Xcalibur™ software (Thermo Fisher Scientific Inc.). Full MS scan data were obtained from *m*/*z* 100 to 1500, with a resolution of 70,000, while data-dependent MS/MS spectra were acquired at a resolution of 35,000 using various collision energies. The proposed chemical structures were determined using Mass Frontier software (version 6.0; HighChem Ltd., Bratislava, Slovakia).

### 4.7. Data Pre-Processing and Multivariate Data Analysis

Overall strategies were adapted from previous reports [39,40,46]. Orbitrap™ MS spectral data were pre-processed with MZmine 2 (available on: http://mzmine.github.io/, version 2.20) [47], which was used to detect mass spectra, build and deconvolute chromatograms, remove isotopes, align chromatograms, and generate a data matrix of ion identity (*m*/*z* and retention time) with abundance. To improve drug metabolite detection, the generated data matrix was manually examined and refined by focusing on excluding isotopes and peak alignment. Principal component analysis (PCA), PLS-DA, and OPLS-DA were conducted using SIMCA (version 14.0; MKS Data Analytics Solutions, Umeå, Sweden). A PCA model was used to obtain an overview of the global data matrix and the PLS-DA and OPLS-DA models were used to maximize the distinction of data set from each experimental group. Data were normalized by Pareto scaling. Variables that significantly contributed to the separation of each group in the PLS-DA or OPLS-DA model were focused on as potential metabolites of KCZ and sorted in accordance with retention time to distinguish metabolite candidates from co-eluent peaks, such as sodium adduct, multiple charged ions, or in-source fragmented ions. The corresponding variables were structurally elucidated using the MS/MS spectra and isotope patterns.

## 5. Conclusions

The identification of metabolites of KCZ in mouse and human was performed using a metabolomics approach. Our understanding of the metabolic fate of KCZ was improved and metabolites associated with hepatotoxicity were suggested. Moreover, mouse is appropriate for assessment of the metabolism and toxicity of KCZ. This approach can be used to fill the gap in our knowledge of the relationship between drug metabolism and drug-induced toxicity.

## Figures and Tables

**Table 1:** Chemical formulae, molecular ions, mass accuracies, retention times, and positive biological samples for ketoconazole and its metabolites.

ID	Formula	[M + H]^+^ (*m*/*z*)		Error (ppm)	*t*_R_ (min)	Identified in	References and Comments
		Theoretical	Observed				
KCZ	C_26_H_28_Cl_2_N_4_O_4_	531.1560	531.1552	−1.56	7.9	-	Parent
M1	C_26_H_30_Cl_2_N_4_O_6_	565.1615	565.1599	−2.85	10.5	MLM, HLM, MHP, HHP, MF	[11]
M2	C_24_H_28_Cl_2_N_4_O_4_	507.1560	507.1554	−1.2	7.9	MLM, HLM, HHP, MF	[11]
M3	C_24_H_22_Cl_2_N_4_O_5_	517.1040	517.1032	−1.64	6.0	MLM, MHP, MF	[11]
M4	C_26_H_28_Cl_2_N_4_O_5_	547.1510	547.1508	−0.24	6.2	MLM, HLM, MHP, HHP, MF	[11]
M5	C_26_H_28_Cl_2_N_4_O_5_	547.1510	547.1502	−1.46	7.3	MLM, HLM, MHP, HHP, MF	[11]
M6	C_26_H_28_Cl_2_N_4_O_5_	547.1510	547.1504	−1.02	7.8	MLM, HLM, MHP, HHP, MF	[11]
M7	C_26_H_28_Cl_2_N_4_O_5_	547.1510	547.1508	−0.24	9.2	MLM, HLM, HHP	[11]
M8	C_24_H_26_Cl_2_N_4_O_4_	505.1404	505.1401	−0.5	5.1	MLM, HLM, MHP, HHP, MF	[11]
M9	C_11_H_10_Cl_2_N_2_O	257.0243	257.0243	+0.08	1.7	MLM, HLM, MHP, HHP, MF	[11]
M10	C_26_H_26_Cl_2_N_4_O_5_	545.1353	545.1349	−0.75	7.0	MLM, HLM, HHP, MF	[11]
M11	C_24_H_26_Cl_2_N_4_O_3_	489.1455	489.1447	−1.68	3.6	MLM, HLM, MHP, HHP, MF	[11]
M12	C_20_H_19_Cl_2_N_3_O_3_	420.0876	420.0878	+0.71	1.8	MLM, MHP, HHP	[11]
M13	C_20_H_19_Cl_2_N_3_O_3_	420.0876	420.0872	−1.1	2.9	MLM, MHP, HHP, MF	[11]
M14	C_14_H_12_Cl_2_N_2_O_4_	343.0247	343.0243	−1.08	4.4	MLM, MF	[11]
M15	C_25_H_26_Cl_2_N_4_O_4_	517.1404	517.1394	−1.91	7.7	MLM	[8]
M16	C_22_H_24_Cl_2_N_4_O_3_	463.1298	463.1293	−1.06	2.9	MLM, HLM, MHP, HHP, MF	[8]
M17	C_24_H_24_Cl_2_N_4_O_4_	503.1247	503.1252	+0.93	5.9	MLM	Novel
M18	C_24_H_24_Cl_2_N_4_O_4_	503.1247	503.1241	−1.31	7.4	MLM, HLM, MHP, HHP, MF	Novel
M19	C_24_H_24_Cl_2_N_4_O_5_	519.1197	519.1204	+1.48	5.8	MLM	Novel
M20	C_26_H_26_Cl_2_N_4_O_4_	529.1404	529.1397	−1.4	7.8	MLM, HLM, MHP, HHP, MF	Novel
M21	C_32_H_36_Cl_2_N_4_O_10_	707.1881	707.1865	−2.28	8.1	MHP, HHP, MF	[13]
M22	C_24_H_28_Cl_2_N_4_O_5_	523.1510	523.1503	−1.3	6.9	MHP, MF	Novel
M23	C_26_H_28_Cl_2_N_4_O_6_	563.1459	563.1450	−1.62	10.2	HHP, MF	[11]
M24	C_26_H_28_Cl_2_N_4_O_6_	563.1459	563.1453	−0.98	10.4	HHP, MF	[11]
M25	C_24_H_28_Cl_2_N_4_O_4_	507.1560	507.1558	−0.49	8.0	HHP, MF	Novel
M26	C_26_H_30_Cl_2_N_4_O_5_	549.1666	549.1663	−0.62	5.5	MF	Novel
M27	C_24_H_26_Cl_2_N_4_O_5_	521.1353	521.1346	−1.38	6.6	MF	Novel
M28	C_24_H_26_Cl_2_N_4_O_6_	537.1302	537.1295	−1.41	7.0	MF	Novel

MLM: mouse liver microsomes; HLM: human liver microsomes; MHP: mouse hepatocytes; HHP: human hepatocytes; MF: mouse feces.

**Table 2:** Chemical formulae, molecular ions, mass accuracies, retention times, and positive biological samples for cyanide adducts of ketoconazole and its metabolites.

Adducts	Formula	[M+H]^+^ (*m*/*z*)		Error (ppm)	*t*_R_ (min)	Identified in	References and Comments
		Theoretical	Observed				
KCZ-CN	C_27_H_27_Cl_2_N_5_O_4_	556.1513	556.1513	−0.07	7.2	MLM, HLM	[11,28,29]
OH-KCZ-CN	C_27_H_27_Cl_2_N_5_O_5_	572.1462	572.1457	−0.89	7.0	MLM, HLM	[11,28,29]
*N*-deacetyl-KCZ-CN	C_25_H_25_Cl_2_N_5_O_3_	514.1407	514.1416	+1.71	6.5	MLM, HLM	Novel

MLM: mouse liver microsomes; HLM: human liver microsomes.

## Supplementary Materials

Supplementary materials can be found at www.mdpi.com/1422-0067/18/3/621/s1.
