# Supplementary material for: Revisiting the Metabolism and Bioactivation of Ketoconazole in Human and Mouse Using Liquid Chromatography–Mass Spectrometry-Based Metabolomics
Source: Int J Mol Sci. 2017 Mar 13;18(3):621. doi: 10.3390/ijms18030621 (PMC5372636; doi:10.3390/ijms18030621)
Supplement: Supplementary file 1 [file ijms-18-00621-s001.pdf]

Supplementary material

Revisiting the metabolism and bioactivation of ketoconazole in mouse and human using liquid chromatography–mass spectroscopy-based metabolomics

Ju-Hyun Kim, Won-Gu Choi, Sangkyu Lee and Hye Suk Lee

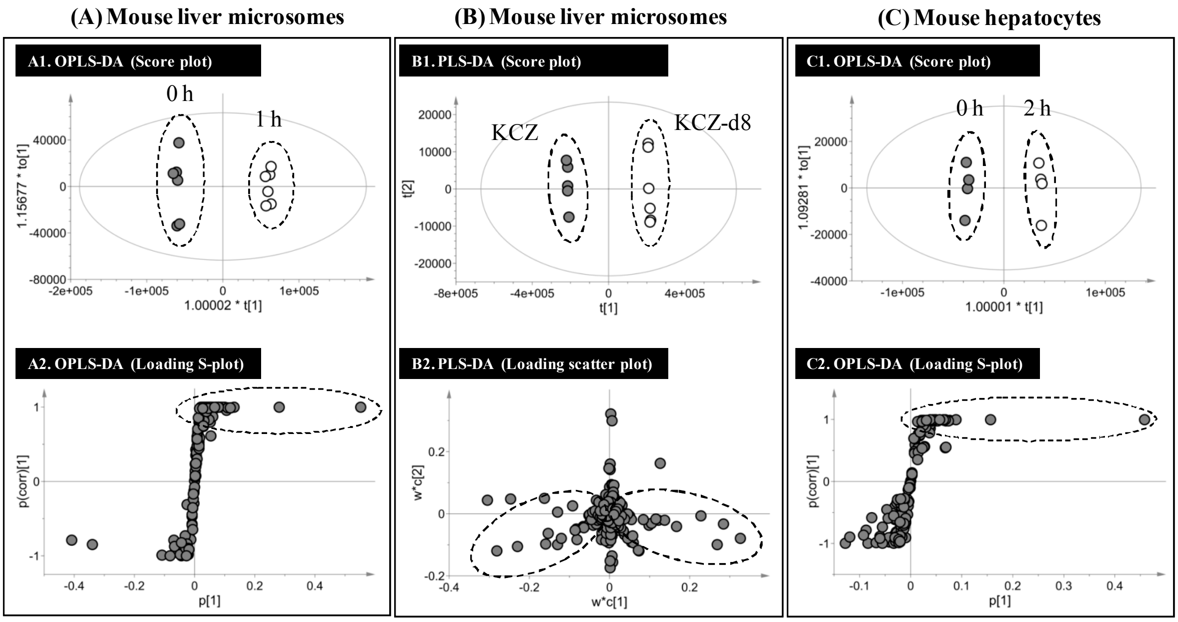

Figure S 1. Multivariate data analysis for the profiling of ketoconaozle metabolites. (A) Score plot and loading S-plot generated by orthogonal partial least squares-discriminant analysis (OPLS-DA) from mouse liver microsomal incubates of ketoconazole. (B) Score plot and loading scatter plot generated by partial least squares-discriminant analysis (PLS-DA) from mouse liver microsomal incubates of ketoconazole and ketoconazole-d8. (C) Score plot and loading S-plot generated by (OPLS-DA) from mouse hepatocytes incubates of ketoconazole.

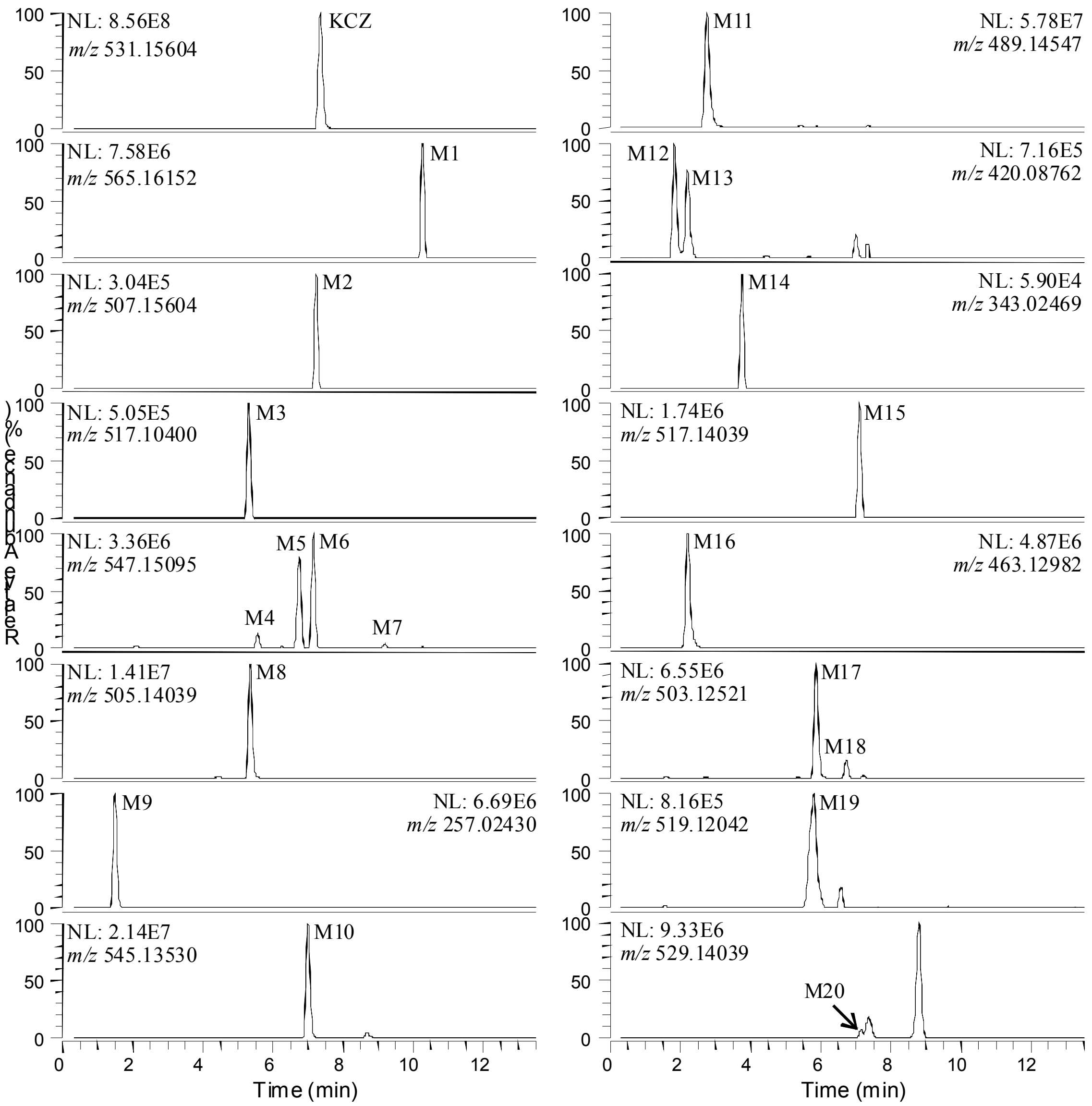

Figure S 2. Extracted ion chromatograms of ketoconazole and its possible metabolites after incubation of 20 μM ketoconazole with mouse liver microsomes for 1 h at 37°C. The chromatograms were extracted with mass accuracy of 3 ppm.

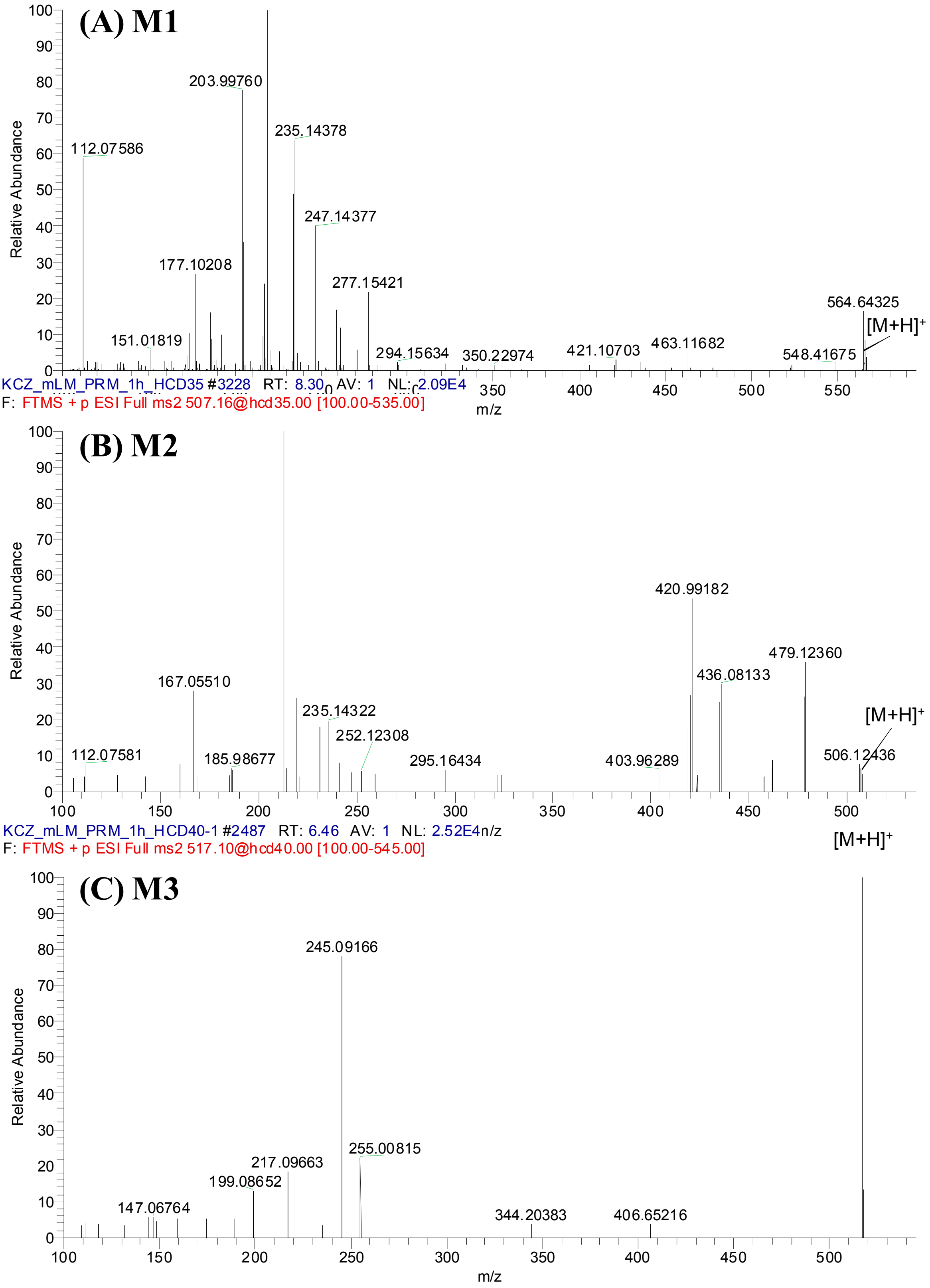

Figure S 3. Product ion spectra of (A) M1, (B) M2, and (C) M3.

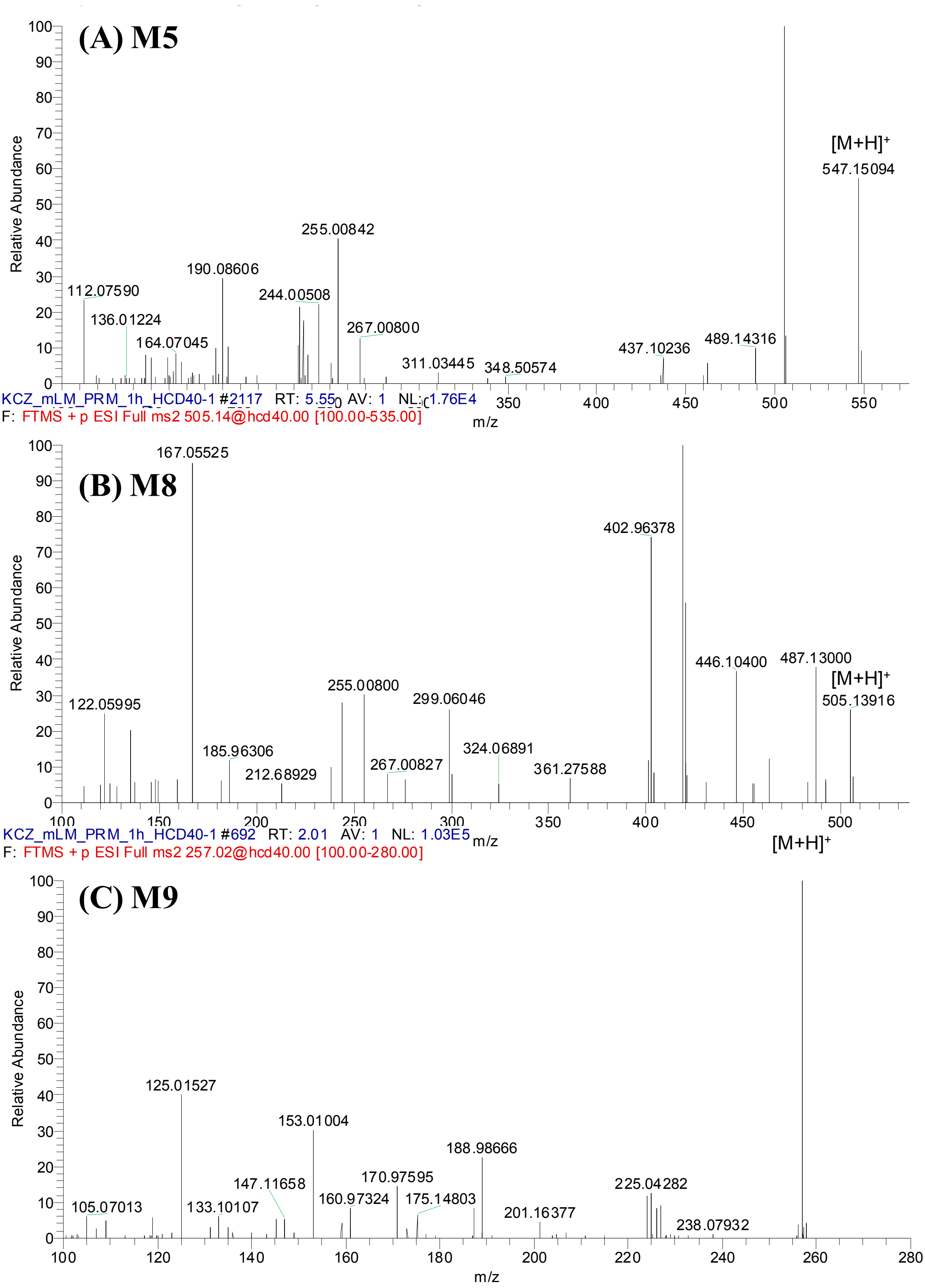

Figure S 4. Product ion spectra of (A) M5, (B) M8, and (C) M9.

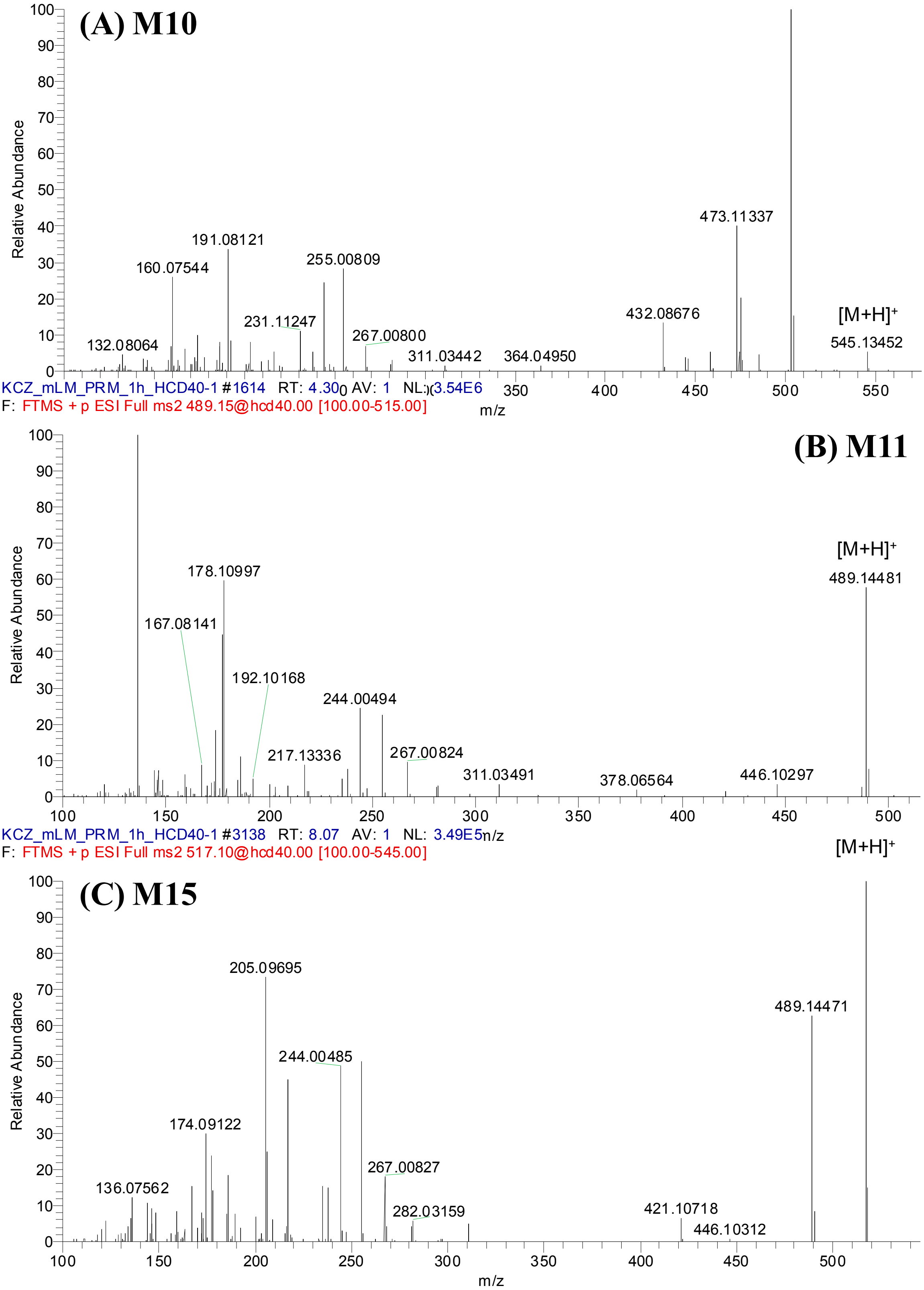

Figure S 5. Product ion spectra of (A) M10, (B) M11, and (C) M15.

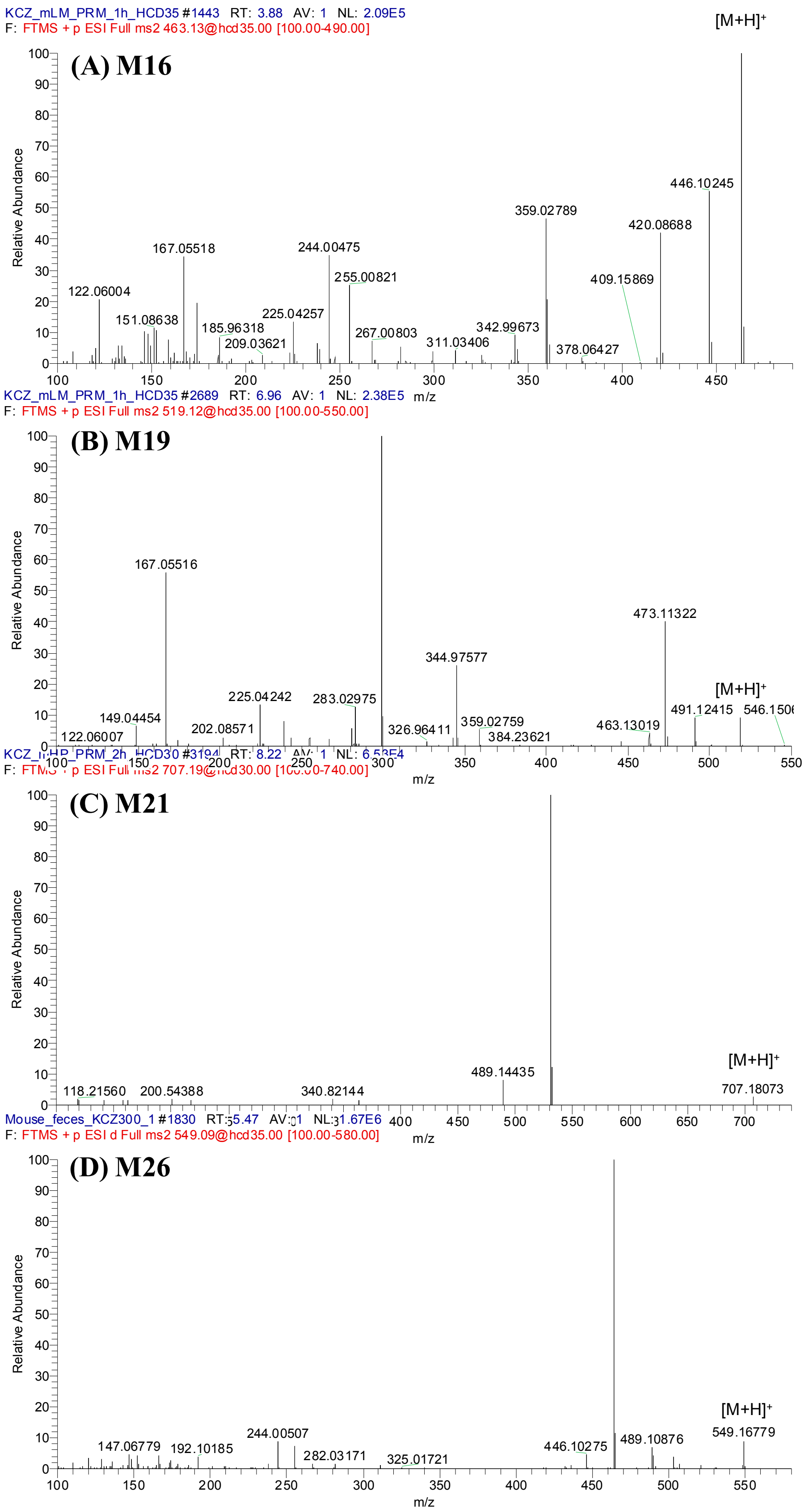

Figure S 6. Product ion spectra of (A) M16, (B) M19, (C) M21, and (D) M26.

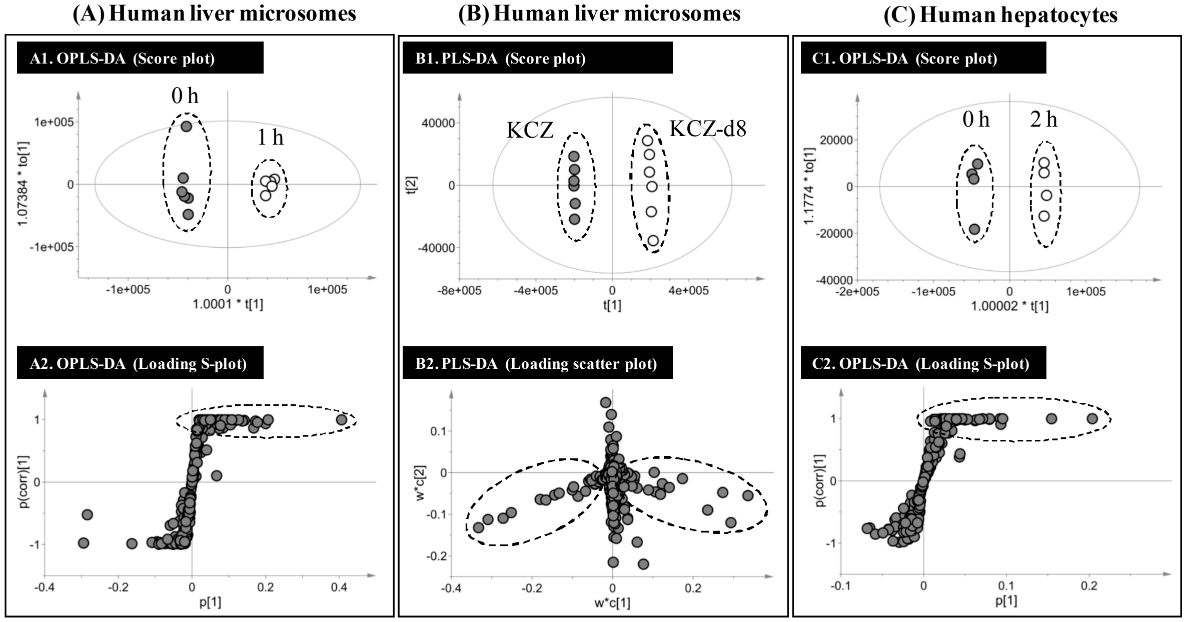

Figure S 7. Multivariate data analysis for the profiling of ketoconaozle metabolites. (A) Score plot and loading S-plot generated by orthogonal partial least squares-discriminant analysis (OPLS-DA) from human liver microsomal incubates of ketoconazole. (B) Score plot and loading scatter plot generated by partial least squares-discriminant analysis (PLS-DA) from human liver microsomal incubates of ketoconazole and ketoconazole-d8. (C) Score plot and loading S-plot generated by (OPLS-DA) from human hepatocytes incubates of ketoconazole.

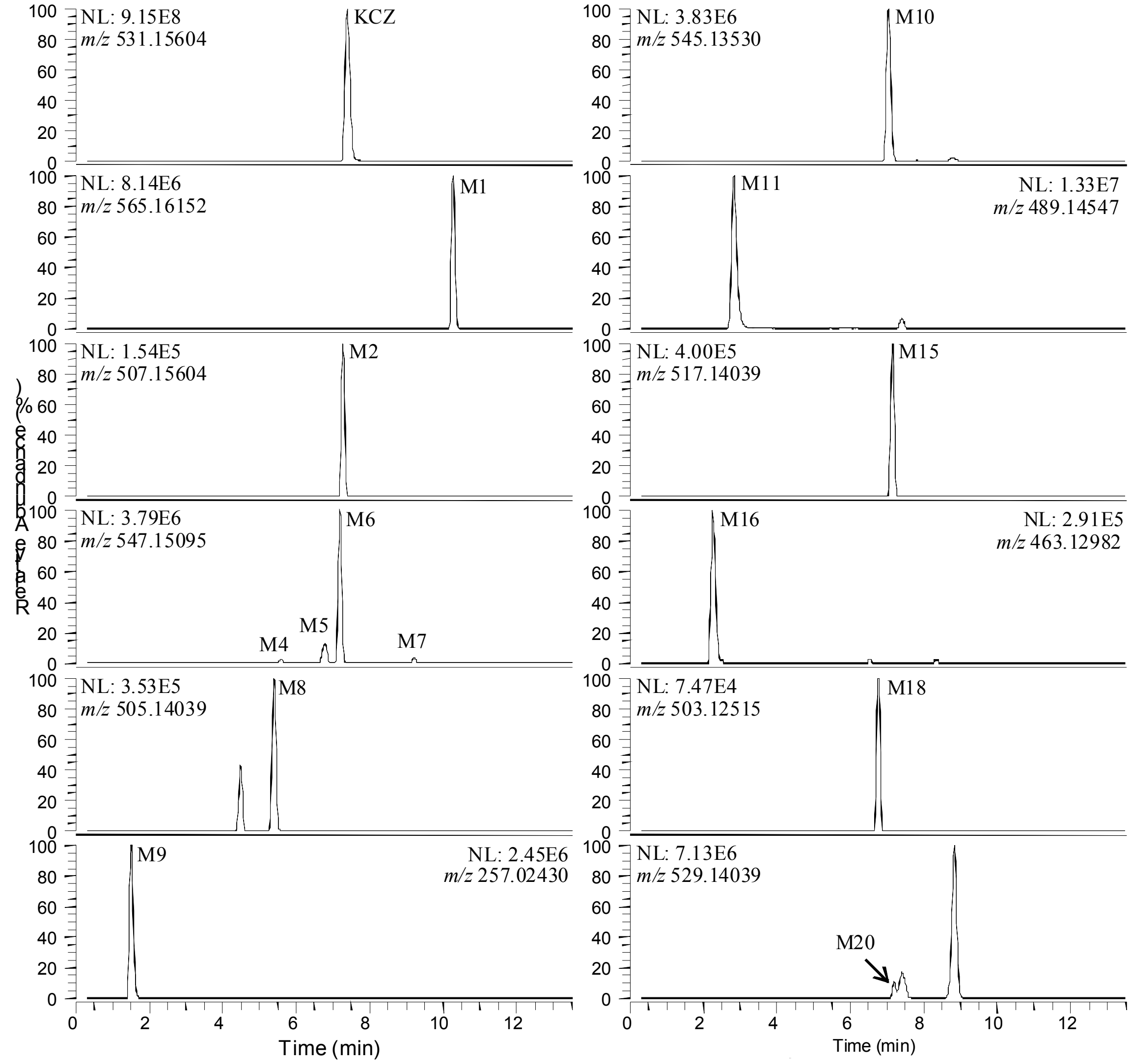

Figure S 8. Extracted ion chromatograms of ketoconazole and its possible metabolites after incubation of 20 µM ketoconazole with human liver microsomes for 1 h at 37°C. The chromatograms were extracted with mass accuracy of 3 ppm.

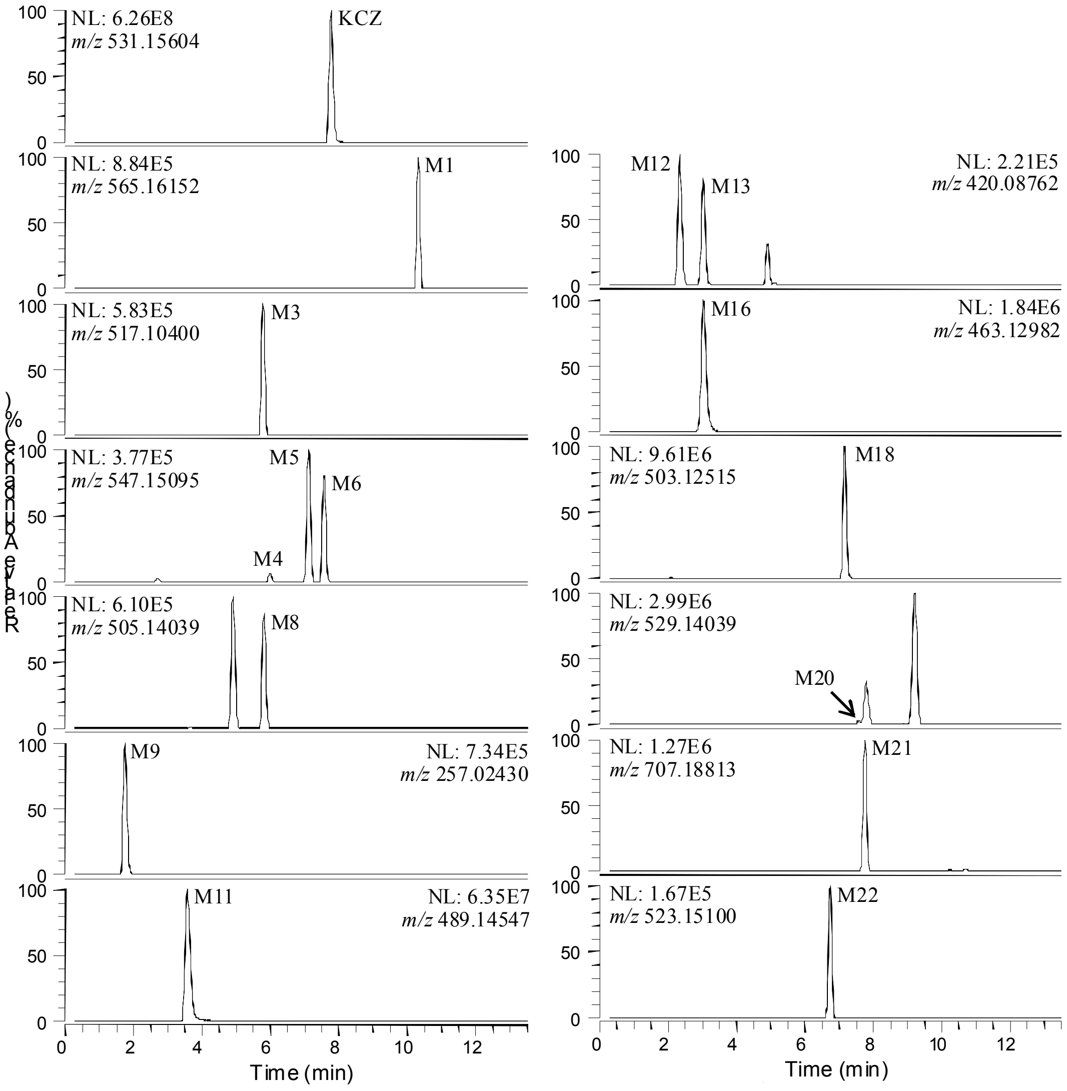

Figure S 9. Extracted ion chromatograms of ketoconazole and its possible metabolites after incubation of 20 µM ketoconazole with mouse hepatocytes for 2 h at 37°C in a humidified CO_2 static incubator. The chromatograms were extracted with mass accuracy of 3 ppm.

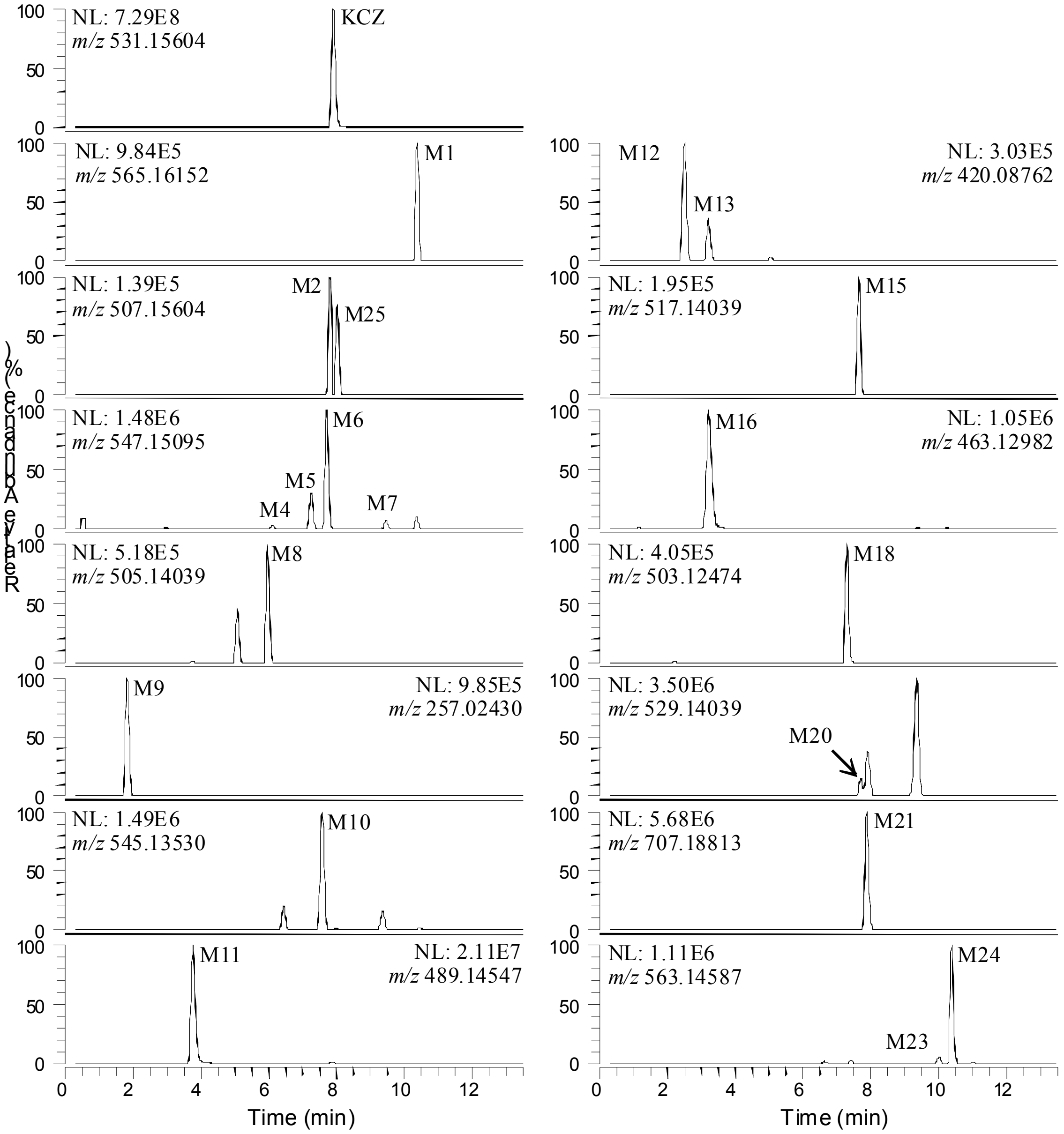

Figure S 10. Extracted ion chromatograms of ketoconazole and its possible metabolites after incubation of 20 µM ketoconazole with human hepatocytes for 2 h at 37°C in a humidified CO_2 static incubator. The chromatograms were extracted with mass accuracy of 3 ppm.
